# Locally advanced rectal cancer transcriptomic-based secretome analysis reveals novel biomarkers useful to identify patients according to neoadjuvant chemoradiotherapy response

**DOI:** 10.1038/s41598-019-45151-w

**Published:** 2019-06-18

**Authors:** Luisa Matos do Canto, Sarah Santiloni Cury, Mateus Camargo Barros-Filho, Bruna Elisa Catin Kupper, Maria Dirlei Ferreira de Souza Begnami, Cristovam Scapulatempo-Neto, Robson Francisco Carvalho, Fabio Albuquerque Marchi, Dorte Aalund Olsen, Jonna Skov Madsen, Birgitte Mayland Havelund, Samuel Aguiar, Silvia Regina Rogatto

**Affiliations:** 1 International Research Center - CIPE, A.C.Camargo Cancer Center, Sao Paulo, 04002-010 Brazil; 2 Department of Clinical Genetics, University Hospital of Southern Denmark, Vejle, 7100 Denmark; 3 Department of Morphology – Institute of Bioscience, São Paulo State University (UNESP), Botucatu, 18618689 Brazil; 4 Department of Pelvic Surgery, A.C.Camargo Cancer Center, Sao Paulo, 04002-010 Brazil; 5 Department of Pathology, A.C.Camargo Cancer Center, Sao Paulo, 04002-010 Brazil; 6 Molecular Oncology Research Center, Barretos, and Diagnósticos da América (DASA), São Paulo, Brazil; 7 Department of Biochemistry and Immunology, University Hospital of Southern Denmark, Vejle, 7100 Denmark; 8 Danish Colorectal Cancer Center South, Vejle, 7100 Denmark; 9 Institute of Regional Health Research, Faculty of Health Sciences, University of Southern Denmark, Vejle, 7100 Denmark; 10 Department of Oncology, University Hospital of Southern Denmark, 7100 Vejle, Denmark

**Keywords:** Tumour biomarkers, Rectal cancer

## Abstract

Most patients with locally advanced rectal cancer (LARC) present incomplete pathological response (pIR) to neoadjuvant chemoradiotherapy (nCRT). Despite the efforts to predict treatment response using tumor-molecular features, as differentially expressed genes, no molecule has proved to be a strong biomarker. The tumor secretome analysis is a promising strategy for biomarkers identification, which can be assessed using transcriptomic data. We performed transcriptomic-based secretome analysis to select potentially secreted proteins using an *in silico* approach. The tumor expression profile of 28 LARC biopsies collected before nCRT was compared with normal rectal tissues (NT). The expression profile showed no significant differences between complete (pCR) and incomplete responders to nCRT. Genes with increased expression (pCR = 106 and pIR = 357) were used for secretome analysis based on public databases (Vesiclepedia, Human Cancer Secretome, and Plasma Proteome). Seventeen potentially secreted candidates (pCR = 1, pIR = 13 and 3 in both groups) were further investigated in two independent datasets (TCGA and GSE68204) confirming their over-expression in LARC and association with nCRT response (GSE68204). The expression of circulating amphiregulin and cMET proteins was confirmed in serum from 14 LARC patients. Future studies in liquid biopsies could confirm the utility of these proteins for personalized treatment in LARC patients.

## Introduction

Rectal cancer is the 8^th^ most incident cancer worldwide, with 704,376 new cases in 2018 and 310,394 deaths in the same period^1^. Patients with locally advanced rectal cancer (LARC) are referred to a multimodal neoadjuvant treatment based on 5-fluorouracil (5-FU) and radiotherapy (nCRT) followed by surgery. This treatment strategy has contributed to reduce the recurrence rates^2^. Moreover, up to 30% of these patients will achieve a pathological complete response (pCR). Complete responders are associated with lower rates of local and distant metastases, and better survival compared to patients with incomplete response (pIR)^3^. Considering the high morbidity of the surgery^4,5^ and the severe side effects of the treatment^6^, it is mandatory to identify patients who will benefit from this treatment. A new approach on LARC management has been proposed using a non-operative “watch and wait” strategy^7^ aiming the organ preservation. As a result, a set of patients will be spared of the side effects, which could be especially relevant in those at early onset, whose incidence of the disease is increasing^8^.

Using transcriptome data analysis, several studies failed in identifying differentially expressed genes with the potential of predicting nCRT response^9,10^. The results reported by different authors were not subsequently validated^11^. The criteria used for the selection of the patients, tumor heterogeneity, different platforms to assess the gene expression and the methods used in the analysis can contribute to the lack of consistent results^9^. Interestingly, three different studies using the same platform (HG-U133, Affymetrix, Santa Clara, CA) were reanalyzed and the final list of differentially expressed genes did not match with the initial results reported by the authors^10^. Nevertheless, gene expression data is a powerful tool to predict potentially secreted proteins^12,13^, which can be used to identify biomarkers of response to nCRT. Genes highly expressed by the tumor can be translated into secreted proteins composing the tumor secretome.

Tumor secretome plays an important role in well-known aspects of cancer, including increased cell proliferation, reduced apoptosis, immunological evasion, angiogenesis, altered energy metabolism, metastasis and development of resistance to therapy^14,15^. Consequently, cancer secretome is useful to identify biomarkers secreted in the blood circulation with potential clinical application^16,17^. For example, vimentin and bone marrow stromal antigen 2 were identified as novel putative colorectal biomarkers using a secretome-based analysis in serum and plasma of patients, respectively^18,19^.

In the last years, microarrays, sequencing, and mass spectrometry have been used for global identification of the secretome components^13,16,17,20^. The development of several bioinformatic tools allow the prediction of secreted proteins based on genomic/transcriptomic annotations and in the interpretation of large-scale data^12^. Secreted proteins can be more easily monitored and detected in the circulation of cancer patients being of great value for clinical practice.

In this study, whole transcriptomic analysis was performed in 28 LARC compared with normal rectal samples to select genes with increased expression according to nCRT response. We coupled the gene expression analysis with *in silico* prediction algorithms to identify the secretory pathways and subcellular localization of the proteins encoded by each gene. Once selected, the list of potentially secreted proteins was used as input to the available secretome-related databases to confirm the presence of the proteins in colorectal cancer (CRC) related samples. As we foresee that these proteins could be detected using liquid biopsies, we also evaluated whether the potential biomarkers have been described in human plasma. A combined information of all these analyses was used to identify potentially secreted proteins, which can be potential biomarkers of nCRT response.

## Results

In our cohort of 28 patients (16 male and 12 women), the median age at diagnosis was 59 years. Nineteen (68%) tumors were classified with moderated cell differentiation and three patients developed metastasis. Eleven (39%) LARC patients achieved pCR and 17 (61%) pIR. Clinical and histopathological information of LARC patients is presented in Table 1.

**Table 1:** Clinical and histopathological features of 42 locally advanced rectal cancer patients (28 evaluated by transcriptome analysis and 14 for protein expression in serum samples).

Characteristics	N=28	N=14
**Median age at diagnosis** (range, years)	59 (26-80)	69.5 (54-81)
**Gender**		
Male	16	8
Female	12	6
**Response to neoadjuvant therapy**		
pCR	11	2
pIR	17	12
**cT stage**		
T2	6	0
T3	20	9
T4	2	5
**cN stage**		
N0	9	0
N+	19	14
**ypT stage**		
T0	12	2
T1	1	0
T2	10	6
T3	3	5
T4	2	1
**ypN stage**		
N0	24	8
N+	4	6
Metastasis	3	0
**Cell Differentiation**		
Well	8	1
Moderate	19	3
NA	1	10

TNM: tumor-node-metastasis, c: clinical and yp: pathological evaluation after neoadjuvant chemotherapy (AJCC 7th edition), pCR: pathological complete response, pIR: pathological incomplete response, NA: not available.

**Figure 1:**
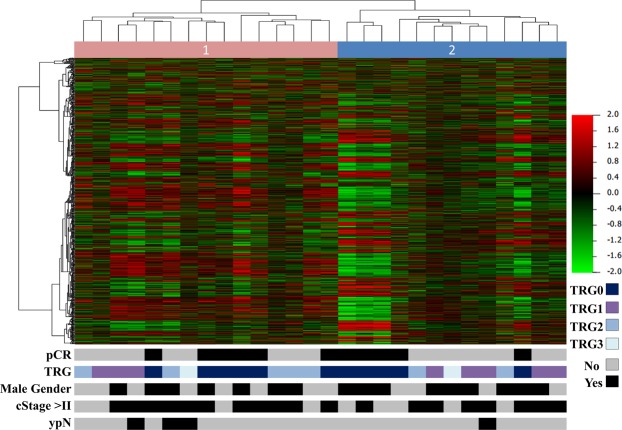
Unsupervised hierarchical clustering analysis of 28 locally advanced rectal carcinomas (LARC) based on the expression values of probes with standard deviation > 0.2 compared with clinical and histopathological features of each patient (bars below in the heatmap). The pink and blue bars represent two clusters (1 and 2, respectively), with no clear differences among gender, clinical stage (cStage), response to therapy, and pathological lymph node status after treatment (ypN). pCR: Pathological Complete Response, TRG: Tumor Regression Grade.

Unsupervised hierarchical clustering analysis of gene expression data (Standard Deviation > 0.2; Euclidian distance and complete linkage) resulted in two groups with no clear distinction according to the clinical and pathological features, including response to nCRT (Fig. 1). A comparison of each group (pCR and pIR) with NT resulted in 446 differentially expressed genes (DEG) in pCR patients, of which 378 were coding genes (106 over- and 272 down-expressed, respectively), and 68 non-coding genes (30 over- and 38 down-expressed, respectively). The comparison between pIR and NT tissues revealed 913 DEG, of which 678 were coding genes (358 over- and 320 down-expressed), and 235 non-coding genes (149 over- and 86 down-expressed). The differentially expressed coding genes identified in pCR and pIR compared with normal tissues are shown in Supplementary Table S1. Microarray data are deposited into the Gene Expression Omnibus (GEO) database (http://www.ncbi.nlm.nih.gov/gds/) with the accession number GSE123390.

Enrichment analysis of the differentially expressed genes (IPA, Ingenuity Pathway Analysis software) revealed alteration of gene expression in 84 canonical pathways in pCR and 88 in pIR. Twenty-seven pathways were exclusively identified in pCR, comprising immune-related pathways, Wnt signaling, cell metabolism, and cell adhesion (Supplementary Table S2). The 31 pathways found exclusively in pIR were related to cell cycle regulation, DNA damage repair, and regulation of gene transcription and translation (Supplementary Table S2).

### Rectal cancer secretome profile according to nCRT response

To identify potentially secreted proteins to be used as biomarkers of response to nCRT in LARC patients, we first obtained the amino acid sequences of translated proteins by the over-expressed coding transcripts in pCR (N = 106) and pIR (N = 358) using UniprotKB database. All but two genes, which encode proteins with more than 4,000 amino acids, were used as input for prediction analysis using the tools available in the Center for Biological Sequence Analysis (CBS) servers. This analysis resulted in 40 and 104 proteins potentially secreted by classical or non-classical pathways in pCR and pIR, respectively (Supplementary Table S3). Twenty nine of 40 proteins or mRNAs identified in pCR and 92 of 104 in pIR were described in extracellular vesicles (EV) from colorectal carcinoma related samples (cell lines, urine, saliva, plasma, tumors or abdominal lavage) (Vesiclepedia/Exocarta).

**Figure 2:**
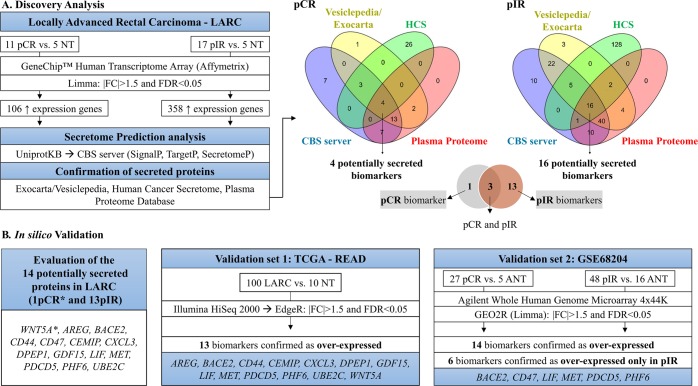
Strategies used for the identification of potentially secreted proteins that could be used as biomarkers of response to neoadjuvant chemoradiotherapy (nCRT) in locally advanced rectal cancer patients (LARC). (**A**) The discovery set was composed of five normal rectal tissues (NT, autopsies) and LARC biopsies from 11 patients with pathological complete response (pCR) and 17 with incomplete response (pIR) to nCRT. The over-expressed genes were used for *in silico* analysis of secreted proteins with the prediction tools available in the CBS server (TargetP, SignalP, and SecretomeP predictions). Several databases (Human Cancer Secretome Database - HCS, Vesiclepedia/Exocarta) were investigated to verify the presence of the potential biomarkers in the secretome of colorectal cancer samples from previously published studies. Plasma Proteome database confirmed the presence of the candidates in the blood circulation. The Venn diagrams illustrate the proteins identified in pCR and pIR using all databases. Four proteins potentially secreted by pCR cases (ERBB3, MMP1, XPO1, and WNT5A) and 16 by pIR (AREG, BACE2, CD44, CD47, CEMIP, CXCL3, DPEP1, ERBB3, GDF15, LIF, MET, MMP1, PDCD5, PHF6, UB2C, and XPO1) were identified in all consulted secretome databases. (**B**) Gene expression data from TCGA-READ and GSE68204 were used for *in silico* validation. Similar analysis used in our dataset was applied for the validation cohort. Among the biomarkers found in the discovery analysis, *CD47* was not detected among the differentially expressed genes in the TCGA cohort. ANT: adjacent normal tissue; LARC: locally advanced rectal cancer; TCGA-READ: The Cancer Genome Atlas-Rectum Adenocarcinoma; FC: Fold Change; FDR: False Discovery Rate; *pCR potentially secreted biomarker.

The Human Cancer Secretome Database (HCSD) presented information of 33 potentially secreted proteins identified in pCR and 152 in pIR cases. Using the Plasma Proteome Database (PPD), 26 predicted proteins for pCR and 73 for pIR were identified as circulating (Supplementary Table S3). By overlapping the information obtained with all databases (CBS server, Vesiclepedia/Exocarta, HCSD and PPD), four potentially secreted proteins associated with pCR and 16 to pIR were found (Fig. 2A). Three proteins (ERBB3, MMP1, and XPO1) were identified in both pCR and pIR groups and were not considered for further analyses. WNT5A was found exclusively in pCR and 13 secreted proteins (AREG, BACE2, CD44, CD47, CEMIP, CXCL3, DPEP1, GDF15, LIF, MET, PDCD5, PHF6, UBE2C) in pIR cases. These potentially secreted proteins were considered as putative biomarkers associated with response to nCRT (Supplementary Table S4).

Using EVpedia, we compared the potential biomarkers with contents of extracellular vesicles from total blood of healthy individuals, serum of CRC patients and two CRC cell lines; one sensitive to 5-FU (HCC2998) and the other resistant (SW620). WNT5A was not found in these samples while UBE2C and CEMIP were observed in the secretome from the 5-FU resistant cell line. CEMIP was also described in the serum of CRC patients.

### *In silico* validation of potential biomarkers of nCRT response

Transcriptomic data of TCGA-READ (The Cancer Genome Atlas – Rectal Adenocarcinoma) were retrieved from 100 LARC compared with 10 ANT revealing 8,717 differentially expressed genes, of which 4,016 were over- and 4,701 were down-expressed. This analysis confirmed increased gene expression levels in 13 of 14 potential biomarkers found in our LARC cases, except *CD47* (Table 2, Fig. 2B).

**Table 2:** Gene expression levels (FC: fold change) of the potentially secreted biomarkers in the discovery and validation set of rectal cancer compared with normal rectal tissues.

Gene Name	Internal		GSE68204		TCGA-READ
	11 pCR vs. 5 NT	17 pIR vs. 5 NT	11 pCR vs. 5 ANT	48 pIR vs. 16 ANT	100 LARC vs. 10 ANT
*WNT5A*	1.58		5.57	10.36	2.84
*AREG*		2.44	4.70	6.73	2.59
*BACE2*		1.54		4.91	2.36
*CD44*		2.00	3.49	4.13	2.30
*CD47*		1.75		2.41	
*CEMIP*		2.26	12.67	22.24	33.89
*CXCL3*		1.83	12.86	10.18	6.16
*DPEP1*		1.52	83.08	66.54	202.00
*GDF15*		2.17	4.24	5.38	4.72
*LIF*		1.60		1.94	3.49
*MET*		1.66		5.45	2.78
*PDCD5*		1.59		2.67	2.04
*PHF6*		1.51		3.98	1.74
*UBE2C*		1.58	4.95	7.09	4.29

pCR: pathological complete response; pIR: pathological incomplete response; NT: normal tissue; ANT: adjacent normal tissue; LARC: locally advanced rectal cancer; TCGA-READ: The Cancer Genome Atlas -Rectum Adenocarcinoma.

The microarray data from GSE68204 revealed 1,091 differentially expressed transcripts in 27 pCR (406 over- and 685 down-expressed compared to 5 adjacent normal tissues, ANT) and 13,621 in 48 pIR (11,933 over- and 1,688 down-expressed in relation to 16 ANT). Similar to our findings, all potential biomarkers were up-regulated in tumors compared to ANT. Moreover, *BACE2*, *CD47*, *LIF*, *MET*, *PDCD5*, and *PHF6* presented increased expression levels exclusively in pIR cases (Table 2, Fig. 2B), while *AREG*, *CD44*, *CEMIP*, *CXCL3*, *DPEP1*, *GDF15*, *UBE2C*, and *WNT5A* presented increased expression levels in tumors from both groups of patients (Table 2, Fig. 2B).

### Serum values of amphiregulin and cMET from LARC patients

Circulating AREG and cMET protein levels were measured in serum from a cohort of 14 LARC patients (Table 1). All samples were collected prior to nCRT treatment. The median values for AREG and cMET were 2.27 pg/mL (range 1.10 to 11.97 pg/mL) and 60103 pg/mL (range 44,299 to 81,814 pg/mL), respectively. Only two cases presented pCR, with no statistically significant difference between the median values of AREG (4.37 pg/mL) and cMET (57,117 pg/mL) compared with 12 pIR cases (2.11 pg/mL and 61,515 pg/mL, respectively) (Table 3).

**Table 3:** Quantification of and cMET and AREG protein in serum from 14 LARC patients.

Sample ID	Response	cMET (pg/mL)	AREG (pg/mL)
1	pCR	58,456	3.2
2	pIR	60,203	1.3
3	pIR	59,707	2.0
4	pIR	81,814	4.2
5	pIR	62,826	1.3
6	pIR	76,472	1.3
7	pIR	55,146	1.5
8	pCR	55,777	5.5
9	pIR	66,889	5.9
10	pIR	58,607	2.4
11	pIR	64,997	1.1
12	pIR	60,002	2.2
13	pIR	44,299	10.0
14	pIR	77,260	12.0

pCR: pathological complete response; pIR: pathological incomplete response. The proteins were measured using the automated Single molecule array (Simoa) HD-1 Analyzer platform.

## Discussion

Gene expression profiling of several tumor types that was deeply investigated has resulted in the identification of molecular signatures, the prediction of recurrence risk, response to therapy and also provided information useful to the treatment decision at the individual level. In CRC, the efforts to identify molecular signatures resulted in four molecular subtypes with distinguishing features^21^. Specifically, in rectal cancer, the number of reports and cases evaluated is limited, which evinces the need to better explore this disease taking into account that colon and rectal cancer have been reported as different entities^22^.

In the present study, we used gene expression arrays performed in LARC samples selected from a large cohort of cases, categorized according to response to therapy and following stringent inclusion criteria. Currently, no biomarkers are used in the clinical practice to differentiate patients with rectal cancer responsive or non-responsive to nCRT. Our main aim was to identify a molecular signature able to differentiate these patients. However, and in agreement with literature, the global expression profile of LARC was not able to distinguish pCR from pIR patients^9,10^. The enrichment analysis of genes differentially expressed in the comparison of each group with normal rectal tissue showed deregulation in genes involved in specific pathways.

Computational analysis of gene expression profile may help to select proteins potentially secreted into the bloodstream of patients, revealing novel tumor response biomarkers^18,19^. The analysis of liquid biopsy is a promising strategy that could be easily applied in the routine with several advantages. This minimally invasive procedure allows repeated investigation of tumor markers, and an evaluation of the tumor landscape with blood drawn. The analysis of molecules in the circulation or in other body fluids (as urine, saliva, cerebrospinal fluid, pleural effusion) has been shown to be a powerful tool for diagnosis, prognosis, to monitor the treatment response, and to detect minimal residual disease while sparing patients of painful or laborious procedures (reviewed by^23^).

The coding genes showing increased expression levels in the comparison between pIR/pCR and NT were used to identify potentially secreted proteins. We found 17 potential candidates that were further validated in independent rectal cancer datasets. Three of 17 genes that encode the potentially secreted candidates (*ERBB3*, *MMP1*, and *XPO1*) were deregulated in all cases (pCR and pIR compared to NT), while one was only altered in pCR and 13 in pIR. Although a comparison according to response to therapy was not performed using the TCGA dataset (not available), the increased expression in 13 of 14 transcripts that encode the potential candidates was confirmed in LARC cases (except *CD47*). Using the GSE68204 data, we confirmed that six transcripts of the potentially secreted candidates (*BACE2*, *CD47*, *LIF*, *MET*, *PDCD5*, *PHF6*) were altered exclusively in pIR cases.

The proteins potentially secreted by tumors from patients with pCR or pIR were evaluated in detail in four databases (Vesiclepedia, ExoCarta, HCSD and PPD). Vesiclepedia is a manually curated database of molecules described in extracellular vesicles, while Exocarta specifically reports molecules found in exosomes. The HCSD comprises information on proteomics data published in the field of cancer secretome and tumor microenvironment, and the PPD is one of the largest resources of serum and plasma proteins. The CRC secretome has been investigated in conditional media of CRC cell lines, peritoneal liquid, urine, stool, and serum/plasma from CRC patients^16,24–28^.

The secretome from 5-FU induced senescent CRC cells altered the behavior of non-treated cells, increasing proliferation and invasiveness^27^. Several cytokines were identified in the secretome of these CRC senescent cells, including IL-8, whose transcripts were also over-expressed in our cases (in both, pCR and pIR). In addition, *CXCL3* identified in our pIR cases was previously reported in CRC and associated with worse overall survival^29^. The authors reported that *CXCL3* over-expression was associated with advanced tumor stage, lymphatic invasion, and distant metastases.

Herein, two proteins, cMET and AREG, were detected in serum from LARC patients. No differences were observed in the levels of these proteins comparing two pCR with 12 pIR patients, probably due to the limited number of cases evaluated. A multicentric study with 81 pre-treatment LARC biopsies described MET protein expression associated with incomplete response to nCRT^30^. In residual LARC from pIR patients, MET high expression levels evaluated after nCRT was associated with worse prognosis^31^. *In vitro* and *in vivo* MET inhibition was shown to sensitize CRC cells to irradiation, suggesting its involvement in resistance to treatment. The co-expression of MET and CD47 proteins was a significant independent prognostic factor in 255 patients with luminal-type primary breast cancer^32^. The higher expression of both markers was associated with overall survival (Hazard Ratio = 4.1, p < 0.002), and CD47 was strongly correlated with lymph node metastasis^32^. Higher expression levels of *MET* and *CD47* in LARC from our pIR patients and in the GSE68204 dataset corroborate their potential as biomarkers. AREG, a member of the EGF family, was reported as having high expression in CRC and was associated with tumor invasion, liver metastasis, and lower survival^33^. Recently, serum levels of AREG higher than 25 pg/mL, evaluated in 120 CRC patients, was significantly associated with liver and peritoneal metastasis^34^. The authors also found high levels of serum AREG associated with distant metastasis, mucinous histological grade, lymphovascular and perineural invasion. In serum from rectal cancer (N = 53) patients, high expression of AREG was detected in 24.5% of cases^34^. In our sample set, two cases presented high levels of AREG (10 and 12 pg/mL) and none of them presented distant metastasis or other clinical pathological features as described by Chayangsu *et al*. (2017)^34^. However, our sample set was restricted to 14 LARC cases.

Increased expression levels of *PHF6* were found exclusively in our pIR patients and previously described in microsatellite-stable CRC^35^. A meta-analysis of genomic, transcriptomic and proteomic data of *PHF6* has shown an up-regulation in several cancer types including in breast and colorectal, suggesting its role as an oncogene^36^. Our results and the analysis of the TCGA and GSE68204 datasets support this hypothesis.

Among the potentially secreted proteins in non-responders LARC patients, UBEC2 and CEMIP were previously described in the secretome of CRC. The cell line SW620, resistant to 5-FU, secreted UBE2C and CEMIP proteins (putative pIR biomarkers) while no expression of these proteins was found in the sensitive CRC cell line (HCC2998) (EVpedia). UBE2C is part of a proteasome complex involved in protein ubiquitination, and its elevated expression has been associated with clinical features related to worse prognosis in several tumor types^37^. Increased expression of *CEMIP* (transcript and protein) was described in CRC and associated with poor 5-year survival^38^. Furthermore, *CEMIP* knockout in human colon cancer cells prevented the formation of xenograft tumors in athymic mice^38^. *UBE2C* and *CEMIP* over-expressions were confirmed in LARC from the TCGA-READ dataset and in responders (FC: 4.95 and 12.67, respectively) and non-responders (FC: 7.09 and 22.24) cases from the GSE68204 dataset. Some differences between the studies, including the type of normal tissue used as control, the microarray platform, and the fact of some patients were treated with oxaliplatin in the GSE68204 cohort, can contribute to some discrepancies observed. However, current literature supports these molecules as associated with worse prognosis and to radiation resistance in CRC cells. The inhibition of UBE2C by NSC697923 promoted *CEMIP* down-regulation, increasing the sensitivity to radiotherapy in the SW480 colon cancer cells. NSC697923 was also associated with *BACE2* down- regulation, another protein identified in our study as associated with pIR and confirmed in the GSE68204 dataset^39^. These proteins should be investigated in liquid biopsies from LARC patients to evaluate their potential as predictive and prognostic biomarkers.

The growth differentiation factor 15 (GDF-15) was identified as a putative biomarker in pIR cases. The secretome of CRC cell lines and serum of CRC patients presented higher expression levels of GDF-15, which were associated with lymph node metastasis^40^ and worse outcome^41^. Increased expression levels of GDF-15 in plasma from CRC patients were associated with the short time of recurrence and reduced overall survival^42^. *In vitro* studies have also demonstrated that GDF-15 is associated with resistance to radiation treatment, which was reversed upon knockdown of the gene^43,44^.

WNT5A (Wnt Family Member 5A) was found as a unique potential secreted biomarker in our pCR patients. In addition, the Wnt/β-catenin signaling and the Planar Cell Polarity (PCP) pathways were altered exclusively in pCR cases. At normal levels, this protein is responsible for maintaining normal processes of development, including cell proliferation, differentiation and migration. However, the deregulation of WNT5A promotes an oncogenic or tumor suppressor effects through canonical and non-canonical signaling pathways^45^. In CRC, the WNT5A protein expression was associated with better prognosis^46^. Increased WNT5A protein expression in colorectal tumor tissues was associated with improved survival^47^, and not correlated with *in vivo* tumorigenesis^48^. Although the effect of 5-FU treatment in colon cancer cells has demonstrated that modulation of WNT5A can lead to increased cell viability^49^, our results showed its increased expression in LARC patients with complete nCRT response. The authors showed that increasing or decreasing WNT5A, the cell viability can change upon 5-FU treatment, suggesting that this gene may be involved in the treatment response^49^.

We reported potential candidates to be investigated in liquid biopsy; however, our study has some limitations. The number of differentially expressed genes found in our expression array analysis (Human Transcriptome Array, HTA 2.0) highly differed from the GSE68204 (Agilent 4 × 44 K). The discrepancies could be explained by the use of different platforms, differences in the normal tissue used in both studies (we used normal rectal tissues obtained from autopsies and the authors used surrounding normal tissue), or paraffin-embedded samples used in our sample set, among others. A recent study has demonstrated concerns of using tumor surrounding normal tissues as a control to identify differentially expressed genes, once they presented characteristics that differentiated them from healthy tissues^50^. Furthermore, formalin-fixed and paraffin-embedded (FFPE) samples may cause nucleic acids degradation^51^ and the transcriptional information could be partially lost; although, still maintaining expression profile similarities with frozen tissues^52,53^. Comparing FFPE colorectal tumor samples with paired frozen samples, Zhu *et al*.^54^ reported similarities but not the identical prognostic signature of 516 genes, even using sufficiently high quality of RNA from FFPE samples^54^. Despite these limitations, we were able to confirm the presence of the putative secreted proteins in rectal cancer samples using public databases. We verified their increased expression in two cohorts of LARC, which reinforces their potential as predictive biomarkers. Furthermore, we were able to detect AREG and cMET proteins in the bloodstream of LARC patients giving additional support for their potential as biomarkers. Unfortunately, the limited number of secreted proteins and cases evaluated precluded a robust statistical analysis.

Overall, potentially secreted proteins were uncovered using gene expression arrays and bioinformatics tools in LARC patients. Two tested proteins, AREG, and cMET, were measured in serum from LARC patients reinforcing our *in silico* protein analysis. The putative secreted proteins herein described should be tested in liquid biopsies from LARC patients prior to treatment to investigate the clinical implications as biomarkers to predict prognosis and response to treatment. With the ability to predict pCR from pIR prior to treatment a significant number of patients might be spared from serious side effects.

## Material and Methods

### Patients

A cohort of 556 patients was diagnosed with LARC at A.C.Camargo Cancer Center and Barretos Cancer Hospital, São Paulo, Brazil, from 2006 to 2015. A set of 33 patients was meticulously selected from this retrospective cohort taken into consideration the following inclusion criteria: 1) patients treated with multimodal neoadjuvant therapy with continuous infusion of 5-fluorouracil or oral capecitabine and radiotherapy (total dose of 50.4 Gy) followed by surgery; 2) patients whose biopsy specimens were collected during colonoscopy prior to pre-operative chemoradiotherapy (nCRT); 3) samples available in the biobanks; (4) biopsies evaluated by two specialized pathologists (MDFSB and CSN); (5) comprehensive clinical, pathological and epidemiological data. We excluded patients with history of previous or synchronous cancer and distant metastasis at diagnosis. Response to nCRT was classified according to the absence (pathological complete response - pCR; ypT0N0) or presence of reminiscent viable tumor cells (pathological incomplete response - pIR) in the surgical specimens. Normal rectal tissues from autopsies were also collected and confirmed as healthy tissues by histopathological evaluation. The Human Research Ethics Committee from both Institutions approved the study (Protocols 1884/14 and 1030/2015, respectively). An additional cohort of 14 patients diagnosed with LARC and treated at Vejle Hospital, Vejle, Denmark, between 2016 and 2017 was selected for protein investigation in serum samples. The study was approved by the Regional Committee on Health Research Ethics for Southern Denmark and the Danish Data Protection Agency (Protocol # S20160097). These cases followed the same inclusion and exclusion criteria described for the cohort of 33 patients. This study was performed in accordance with the guidelines and regulations of the above mentioned Ethics Committees, and written informed consent was obtained from all participants or family members (autopsies) prior to sample collection.

The workflow used for the identification of potentially secreted response biomarkers in locally advanced rectal cancer patients according to response to nCRT is depicted in Fig. 2.

### Whole gene expression analysis

Transcriptome analysis of 33 FFPE LARC biopsies and five FFPE histopathologically normal rectum samples were assessed using the high-resolution platform GeneChip™ Human Transcriptome Array 2.0 (HTA, Affymetrix/ThermoFisher, USA). Briefly, RNA was isolated using the RecoverAll™ Total Nucleic Acid Isolation Kit for FFPE (Invitrogen/ThermoFisher, EUA). Amplification and cDNA labeling were performed using the SensationPlus™ FFPE Amplification and WT Labeling Kit assay (Affymetrix/ThermoFisher, USA), according to manufacturer instructions. Three samples yielded insufficient cDNA and were excluded. The final solution was hybridized onto the GeneChip® Probe Array (49-format) for 16 hours at 47 °C and 60 rpm. The staining and washes were automated carried out using the GeneChip Fluidics Station 450 (Affymetrix/ThermoFisher, USA). The scanning was performed using the Affymetrix GeneChip Scanner 7000 (Affymetrix/ThermoFisher, USA).

The CEL files were generated by Affymetrix® GeneChip® Command Console® (AGCC) 4.0. Inter-arrays quantile normalization was performed separately for coding and non-coding RNAs using the Human Genome Organization (HUGO) annotation (https://www.genenames.org/). Two samples presented extremely low overall probe signal and were excluded from further analysis. The comparison between 28 LARC (11 pCR or 17 pIR) with five normal tissues (NT) was performed using the package limma^55^ available for R software (https://www.rproject.org/). The differentially expressed probes in each comparison (pCR *versus* NT and pIR *versus* NT) were selected using False Discovery Ratio (FDR) < 0.05 and |Fold Change (FC)| > 1.5. The lists of differentially expressed genes (DEG) found in pCR and pIR compared with NT were used for pathway enrichment using Ingenuity Pathway Analysis software (IPA, Qiagen).

### Transcriptome-based secretome analysis

An *in silico* approach was used to identify potentially secreted proteins as biomarkers from LARC patients. The over-expressed genes obtained from the transcriptome analysis comparing normal tissues with pCR and pIR cases were selected. The FASTA sequence of the proteins translated by those genes was acquired (UniprotKB; http://www.uniprot.org)^56^ and used for prediction of the subcellular location (TargetP 1.1)^57^, signal peptide and cleavage sites for proteins secreted by classical (SignalP 4.1)^58^ and non-classical secretion pathways (SecretomeP 2.0)^59^. These tools are available in the CBS server (http://www.cbs.dtu.dk/services/). The list of proteins identified as secreted by classical and non-classical secretion pathways and not addressed to the mitochondria was selected for a detailed investigation in databases of secreted proteins in vesicles: ExoCarta (http://www.exocarta.org/)^60^, Vesiclepedia (http://www.microvesicles.org/)^61^, The Human Cancer Secretome Database (HCSD, http://www.cancersecretome.org/)^62^. These databases (Vesiclepedia, Exocarta, HCSD) curate secreted protein-level results from experiments already published in the literature involving different sample types and were used to confirm the presence of proteins in CRC samples. As we foresee that these proteins could be detected using liquid biopsies, we also evaluated whether the potential biomarkers were already described in human plasma using the Plasma Proteome Database (PPD, http://plasmaproteomedatabase.org)^63^. The set of proteins overlapped in all databases were selected as potential biomarkers of pCR or pIR and illustrated using the Venn diagrams (Venny 2.1, http://bioinfogp.cnb.csic.es/tools/venny/). Proteins identified in both lists (pCR and pIR) were excluded from further analysis. EVpedia (http://evpedia.info/)^64^ was used to obtain information of the secretome in CRC cell lines responsive (HCC2998) or resistant (SW620) to 5-FU (http://colonatlas.org/)^65^.

### Validation datasets

Independent datasets of rectal cancer patients were used to confirm the increased expression of the potential biomarkers and their association with response to nCRT. RNA sequencing (Illumina Hiseq 2000 v2) data from adjacent normal tissues and 100 LARC tissues included in the TCGA-READ (consulted in September 2018) were downloaded and pre-processed using the package TCGAbiolinks^66^. The differentially expressed genes were assessed using the default settings of EdgeR package, which included the normalization with Trimmed Mean of M (TMM) and comparison of expression values using exact T-test with False Discovery Ratio correction (FDR)^67^ (available in the R software). The LARC patients from the TCGA dataset have no information on the treatment response.

Large-scale gene expression datasets of LARC having information of the neoadjuvant treatment response available for re-analysis at the Gene Expression Omnibus (GEO: https://www.ncbi.nlm.nih.gov/geo/) repository were used for further comparisons. Studies with no comparisons between tumor and normal tissues or that used a different treatment strategy than those applied in our study were excluded (Supplementary Table S5). Only one study (GSE68204) fulfilled these criteria. The authors provided expression data from adjacent normal tissues (ANT; N = 21) and LARC (N = 59) from patients treated with nCRT^68^. Tumor response was assessed according to the modified tumor regression grade (TRG) classification^69^. Patients were divided in pCR (TRG1) and pIR (TRG2-5). The Whole Human Genome Oligo microarray platform 4 × 44 K (Agilent Technologies, Santa Clara, CA) data from the GSE68204 dataset were analyzed using the default settings of GEO2R tool. The adjusted p values (adj. p) were applied using Benjamini and Hochberg false discovery rate (FDR)^70^ to compare 5 ANT *versus* 11 pCR, and 16 ANT *versus* 48 pIR patients. Genes with FDR < 0.05 and FC > 1.5 were selected as up-regulated for both validation sets.

### Detection of AREG and cMET proteins in serum from LARC patients

Serum collected from 14 LARC patients was used to detect Amphiregulin and cMET proteins. To measure amphiregulin in the serum samples, we used an in-house three-plex assay developed and performed on the automated Single molecule array (Simoa) HD-1 Analyzer platform (Quanterix©, Lexington, MA, USA). The method measures the EGFr ligands amphiregulin, betacellulin and TGF-α simultaneously and have been described in detail in Olsen *et al*.^71^ The capture antibodies used for the three-plex assay were amphiregulin (cat.no. AF262, R&D Systems, Minneapolis, MN, USA), betacellulin (cat.no. AF-261-NA, R&D Systems) and TGF-α (cat.no. AF-239-NA, R&D Systems). The concentration of the detection antibodies was 0.4 µg/mL amphiregulin (cat.no. BAF262, R&D Systems), 0.2 µg/mL betacellulin (cat.no. BAF261, R&D Systems) and 0.1 µg/mL TGF-α (cat.no. BAF239, R&D Systems). The samples were diluted 3-fold in diluent A (Quanterix) with 10 µg/mL Superchemiblock Heterophile Blocking Agent (EMD Millipore, Darmstadt, Germany) and run in duplicates. In-house serum pools were used as controls and included in duplicates in each assay. The total coefficient of variation (CV) was 21% at level 0.8 pg/mL and 12–16% at four different levels ranging between 2 pg/mL and 90 pg/mL. A commercially available Discovery Kit (cat.no. 102073, Quanterix) for the Simoa was used to quantify c-MET in serum samples. The reaction run in duplicates including the calibrators, samples and two controls, according to the manufacturer’s recommendations. The total CV was < 10%.

### Accession number

Data were submitted to Gene Expression Omnibus repository, accession number GSE123390.

## Supplementary information

Supplementary Table S1

Supplementary Table S2

Supplementary Table S3

Supplementary Tables S4 and S5
