# Supplementary material for: Locally advanced rectal cancer transcriptomic-based secretome analysis reveals novel biomarkers useful to identify patients according to neoadjuvant chemoradiotherapy response
Source: Sci Rep. 2019 Jun 18;9:8702. doi: 10.1038/s41598-019-45151-w (PMC6582145; doi:10.1038/s41598-019-45151-w)
Supplement: Supplementary file 4 — Supplementary Tables S4 and S5 [file 41598_2019_45151_MOESM4_ESM.docx]

**Locally advanced rectal cancer transcriptomic-based secretome analysis reveals novel biomarkers useful to identify patients according to neoadjuvant chemoradiotherapy response**. CANTO LM, CURY SS, BARROS-FILHO MC, KUPPER BEC, BEGNAMI MDFS, SCAPULATEMPO-NETO C, CARVALHO RF, MARCHI FA, OLSEN DA, MADSEN JS, HAVELUND BM, AGUIAR JR. S, ROGATTO SR.

**Supplementary Table S4.** Proteins identified as potentially secreted by tumors from patients with locally advanced rectal cancer from our study according to response to neoadjuvant therapy. Proteins identified specifically in each group or shared by both groups, and their relevance for colorectal cancer or other tumor types are described.

| **Symbol** | **Protein Name** | **Sample type** | **Relevance** | **Reference** |
| --- | --- | --- | --- | --- |
| **Pathological Complete Response (pCR)** | | | | |
| WNT5A | Wnt Family Member 5A | CRC patients (n=84) | Protein high expression was correlated with improved patient survival. | Mehdawi, et al. 2016 ^1^ |
|  |  | Colon cancer cell line and mouse model | Increased gene expression induced colon cancer cell migration and invasion (*in vitro)*, but *in vivo* no correlation with tumorigeneses was found*.* | Bakker, et al. 2013 ^2^ |
| **Pathological Incomplete Response (pIR)** | | | | |
| AREG | Amphiregulin | CRC patients (n= 120/53 ReCa) | High protein serum levels were correlated with worse prognosis. | Chayangsu, et al. 2017 ^3^ |
|  |  | CRC patients (n= 226/87 ReCa) | Powerful blood-based biomarker in predicting the presence of CRC. | Chen, et al. 2017 ^4^ |
| BACE2 | Beta-Secretase 2 | Prostate cancer animal model | Reduced expression correlated with low biochemical recurrence | Linn, et al. 2016 ^5^ |
| CD44 | CD44 Molecule | ReCa (n=52) | Independent prognostic factor for recurrence and overall survival. | Saigusa, et al. 2012 ^6^ |
|  |  | ReCa (n=123) | Increased gene expression might be predictive of poor tumor regression after preoperative chemoradiation. | Huh, et al. 2014 ^7^ |
| CD47 | CD47 Molecule | Ovarian cancer (n=223) | Protein over-expression associated with poor prognosis. | Li, et al. 2017 ^8^ |
| CEMIP | Cell Migration Inducing Hyaluronidase 1 | CRC cell lines | Knockdown suppressed proliferation and induced apoptosis. | Liang, et al. 2018 ^9^ |
|  |  | CRC (n=73) | Gene over-expression was associated with poor survival. | Fink, et al. 2015 ^10^ |
| CXCL3 | C-X-C Motif Chemokine Ligand 3 | CRC (n=97) | High gene expression levels associated with risk to develop metastasis. | Doll, et al. 2010 ^11^ |
|  |  | CRC patients (n=498/136ReCa) | High gene expression correlated with worse prognosis | Xiong, et al. 2017 ^12^ |
| DPEP1 | Dipeptidase 1 | CRC (n=78) | High gene expression was associated with lymph node metastasis. | Tachibana, et al. 2017 ^13^ |
|  |  | CRC (n=47/18 ReCa) | Protein over-expressed in high-grade tumors compared with low grade. Recognized as an independent prognostic factor. | Eisenach, et al. 2013 ^14^ |
|  |  | CRC (n=22) | Protein over-expression in CRC compared to normal adjacent | Hao, et al. 2017 ^15^ |
| LIF | Leukemia Inhibitory Factor | Oesophageal adenocarcinoma cell line | High serum/plasma levels associated with treatment resistance. | Buckley, et al. 2018 ^16^ |
|  |  | C26 CRC cells | Driver of cancer cachexia. | Jackman, et al. 2017 ^17^ |
| GDF15 | Growth differentiation factor 15 | CRC patients (n=266/83 ReCa) | High protein expression levels in tissue and in plasma are associated with recurrence and poor survival. | Wallin, et al. 2011 ^18^ |
|  |  | CRC patients (n=215/96 ReCa) | High protein expression levels in tissue and serum are associated with metastasis. | Li, et al. 2016 ^19^ |
|  |  | CRC patients (n=144/66 ReCa) | Serum biomarker of metastasis. | Xue, et al. 2010 ^20^ |
| MET | MET Proto-Oncogene, Receptor Tyrosine Kinase | ReCa (n=52) | High gene expression levels are associated with relapse-free survival. | Kawamura, et al. 2012 ^21^ |
|  |  | CRC (n=102/20 ReCa) | High protein expression associated with disease progression | Gayyed, et al. 2015 ^22^ |
| PDCD5 | Programmed Cell Death 5 | Colon cancer cell lines | Gene and protein over-expression associated with apoptosis. | Yin, et al. 2010 ^23^ |
| PHF6 | PHD Finger Protein 6 | CRC from databases | Increase gene and protein expression in CRC, putative oncogene | Hajjari, et al. 2016 ^24^ |
| UBE2C | Ubiquitin Conjugating Enzyme E2 C | Advanced CRC (n=27) | Gene over-expression associated with tumor progression | Takahashi, et al. 2006 ^25^ |
| **Biomarkers detected in both groups (pCR and pIR)** | | | | |
| ERBB3 | Erb-B2 Receptor Tyrosine Kinase 3 | CRC (n=1716) | Increased protein expression associated with poor prognosis. | Yan, et al. 2018 ^26^ |
| MMP1 | Matrix Metallopeptidase 1 | CRC (n=930) | Prognostic biomarker associated with survival. | Huang, et al. 2018 ^27^ |
| XPO1 | Exportin 1 | CRC cell lines | Inhibition improved the radiation sensitivity. | Ferreiro-Neira, et al. 2016 ^28^ |

CRC:Colorectal cancer; ReCa: Rectal Cancer; C26: Colon-26 Carcinoma Tumor-bearing Mouse; n: number of cases

**References**

1 Mehdawi, L. M., Prasad, C. P., Ehrnstrom, R., Andersson, T. & Sjolander, A. Non-canonical WNT5A signaling up-regulates the expression of the tumor suppressor 15-PGDH and induces differentiation of colon cancer cells. *Mol Oncol* **10**, 1415-1429, doi:10.1016/j.molonc.2016.07.011 (2016).

2 Bakker, E. R. *et al.* Wnt5a promotes human colon cancer cell migration and invasion but does not augment intestinal tumorigenesis in Apc1638N mice. *Carcinogenesis* **34**, 2629-2638, doi:10.1093/carcin/bgt215 (2013).

3 Chayangsu, C., Khunsri, S., Sriuranpong, V. & Tanasanvimon, S. The correlations between serum amphiregulin and other clinicopathological factors in colorectal cancer. *J Gastrointest Oncol* **8**, 980-984, doi:10.21037/jgo.2017.08.15 (2017).

4 Chen, H. *et al.* Development and validation of a panel of five proteins as blood biomarkers for early detection of colorectal cancer. *Clin Epidemiol* **9**, 517-526, doi:10.2147/CLEP.S144171 (2017).

5 Linn, D. E., Penney, K. L., Bronson, R. T., Mucci, L. A. & Li, Z. Deletion of Interstitial Genes between TMPRSS2 and ERG Promotes Prostate Cancer Progression. *Cancer Res* **76**, 1869-1881, doi:10.1158/0008-5472.CAN-15-1911 (2016).

6 Saigusa, S. *et al.* Clinical significance of LGR5 and CD44 expression in locally advanced rectal cancer after preoperative chemoradiotherapy. *Int J Oncol* **41**, 1643-1652, doi:10.3892/ijo.2012.1598 (2012).

7 Huh, J. W., Lee, J. H. & Kim, H. R. Pretreatment expression of 13 molecular markers as a predictor of tumor responses after neoadjuvant chemoradiation in rectal cancer. *Ann Surg* **259**, 508-515, doi:10.1097/SLA.0b013e31829b3916 (2014).

8 Li, Y. *et al.* Overexpression of CD47 predicts poor prognosis and promotes cancer cell invasion in high-grade serous ovarian carcinoma. *Am J Transl Res* **9**, 2901-2910 (2017).

9 Liang, G., Fang, X., Yang, Y. & Song, Y. Knockdown of CEMIP suppresses proliferation and induces apoptosis in colorectal cancer cells: downregulation of GRP78 and attenuation of unfolded protein response. *Biochem Cell Biol* **96**, 332-341, doi:10.1139/bcb-2017-0151 (2018).

10 Fink, S. P. *et al.* Induction of KIAA1199/CEMIP is associated with colon cancer phenotype and poor patient survival. *Oncotarget* **6**, 30500-30515, doi:10.18632/oncotarget.5921 (2015).

11 Doll, D. *et al.* Differential expression of the chemokines GRO-2, GRO-3, and interleukin-8 in colon cancer and their impact on metastatic disease and survival. *Int J Colorectal Dis* **25**, 573-581, doi:10.1007/s00384-010-0901-1 (2010).

12 Xiong, Y., You, W., Wang, R., Peng, L. & Fu, Z. Prediction and Validation of Hub Genes Associated with Colorectal Cancer by Integrating PPI Network and Gene Expression Data. *Biomed Res Int* **2017**, 2421459, doi:10.1155/2017/2421459 (2017).

13 Tachibana, K. *et al.* Clinicopathological examination of dipeptidase 1 expression in colorectal cancer. *Biomed Rep* **6**, 423-428, doi:10.3892/br.2017.870 (2017).

14 Eisenach, P. A. *et al.* Dipeptidase 1 (DPEP1) is a marker for the transition from low-grade to high-grade intraepithelial neoplasia and an adverse prognostic factor in colorectal cancer. *Br J Cancer* **109**, 694-703, doi:10.1038/bjc.2013.363 (2013).

15 Hao, J. J. *et al.* Comprehensive Proteomic Characterization of the Human Colorectal Carcinoma Reveals Signature Proteins and Perturbed Pathways. *Sci Rep* **7**, 42436, doi:10.1038/srep42436 (2017).

16 Buckley, A. M. *et al.* Leukaemia inhibitory factor is associated with treatment resistance in oesophageal adenocarcinoma. *Oncotarget* **9**, 33634-33647, doi:10.18632/oncotarget.25950 (2018).

17 Jackman, R. W. *et al.* Continuous Release of Tumor-Derived Factors Improves the Modeling of Cachexia in Muscle Cell Culture. *Front Physiol* **8**, 738, doi:10.3389/fphys.2017.00738 (2017).

18 Wallin, U. *et al.* Growth differentiation factor 15: a prognostic marker for recurrence in colorectal cancer. *Br J Cancer* **104**, 1619-1627, doi:10.1038/bjc.2011.112 (2011).

19 Li, C. *et al.* Growth differentiation factor 15 is a promising diagnostic and prognostic biomarker in colorectal cancer. *J Cell Mol Med* **20**, 1420-1426, doi:10.1111/jcmm.12830 (2016).

20 Xue, H. *et al.* Identification of serum biomarkers for colorectal cancer metastasis using a differential secretome approach. *J Proteome Res* **9**, 545-555, doi:10.1021/pr9008817 (2010).

21 Kawamura, M. *et al.* Correlation of MACC1 and MET expression in rectal cancer after neoadjuvant chemoradiotherapy. *Anticancer Res* **32**, 1527-1531 (2012).

22 Gayyed, M. F., Abd El-Maqsoud, N. M., El-Hameed El-Heeny, A. A. & Mohammed, M. F. c-MET expression in colorectal adenomas and primary carcinomas with its corresponding metastases. *J Gastrointest Oncol* **6**, 618-627, doi:10.3978/j.issn.2078-6891.2015.072 (2015).

23 Yin, A., Jiang, Y., Zhang, X., Zhao, J. & Luo, H. Transfection of PDCD5 sensitizes colorectal cancer cells to cisplatin-induced apoptosis in vitro and in vivo. *Eur J Pharmacol* **649**, 120-126, doi:10.1016/j.ejphar.2010.09.040 (2010).

24 Hajjari, M., Salavaty, A., Crea, F. & Kee Shin, Y. The potential role of PHF6 as an oncogene: a genotranscriptomic/proteomic meta-analysis. *Tumour Biol* **37**, 5317-5325, doi:10.1007/s13277-015-4250-0 (2016).

25 Takahashi, Y. *et al.* Detection of aberrations of ubiquitin-conjugating enzyme E2C gene (UBE2C) in advanced colon cancer with liver metastases by DNA microarray and two-color FISH. *Cancer Genet Cytogenet* **168**, 30-35, doi:10.1016/j.cancergencyto.2005.12.011 (2006).

26 Yan, Q. *et al.* Association between the overexpression of Her3 and clinical pathology and prognosis of colorectal cancer: A meta-analysis. *Medicine (Baltimore)* **97**, e12317, doi:10.1097/MD.0000000000012317 (2018).

27 Huang, Z., Yang, Q. & Huang, Z. Identification of Critical Genes and Five Prognostic Biomarkers Associated with Colorectal Cancer. *Med Sci Monit* **24**, 4625-4633, doi:10.12659/MSM.907224 (2018).

28 Ferreiro-Neira, I. *et al.* XPO1 Inhibition Enhances Radiation Response in Preclinical Models of Rectal Cancer. *Clin Cancer Res* **22**, 1663-1673, doi:10.1158/1078-0432.CCR-15-0978 (2016).

**Supplementary Table S5**. Gene expression studies in rectal cancer samples according to treatment response available in the Gene Expression Omnibus (GEO) repository not included in our analysis.

| **GEO number** | **Platform** | **Number of Cases** | **Treatment** | **Reference** |
| --- | --- | --- | --- | --- |
| GSE3493 | Human Genome U95 Version 2 Array (Affymetrix) | 46 cases  - | Radiotherapy | 1 |
| GSE35452 | Human Genome U133 Plus 2.0 Array (Affymetrix) | 24R and 22NR | Radiotherapy | 2 |
| GSE53781 | CodeLink Human Whole Genome Array (GE Healthcare) | 10R and 16NR | nCRT (capecitabine or capecitabine and oxaliplatine) | 3 |
| GSE60331 | PrimeView Human Gene Expression Array (Affymetrix) | 7R and 9NR | nCRT (bevacizumab in combination with capecitabine) | 4 |
| GSE46862 | Human Gene 1.0 ST Array (Affymetrix) | 10MI, 36MO, 13NT, 18TO | nCRT | 5 |
| GSE93375 | PrimeView Human Gene Expression Array (Affymetrix) | 5R and 8NR | nCRT (oxaliplatin and fluoropyrimidines) | 6 |

Studies classified patients according to response to the specific treatments into R: responders; NR: non-responders; MI: minimal response; MO: moderate response; NT: near total response; TO: total response; nCRT: neoadjuvant chemoradiotherapy.

**References**

1 Watanabe, T. *et al.* Prediction of sensitivity of rectal cancer cells in response to preoperative radiotherapy by DNA microarray analysis of gene expression profiles. *Cancer Res* **66**, 3370-3374, doi:10.1158/0008-5472.CAN-05-3834 (2006).

2 Watanabe T, Kobunai T, Hashimoto E. Prediction of sensitivity of rectal cancer cells in response to preoperative chemoradiotherapy by DNA microarray analysis of gene expression profiles. Public on Jan 29, 2013. GSE35452. Study not published.

3 Palma, P. *et al.* Expression profiling of rectal tumors defines response to neoadjuvant treatment related genes. *PLoS One* **9**, e112189, doi:10.1371/journal.pone.0112189 (2014).

4 Verstraete, M. *et al.* Combining bevacizumab and chemoradiation in rectal cancer. Translational results of the AXEBeam trial. *Br J Cancer* **112**, 1314-1325, doi:10.1038/bjc.2015.93 (2015).

5 Gim, J. *et al.* Predicting multi-class responses to preoperative chemoradiotherapy in rectal cancer patients. *Radiat Oncol* **11**, 50, doi:10.1186/s13014-016-0623-9 (2016).

6 Goncalves-Ribeiro, S. *et al.* Prediction of pathological response to neoadjuvant treatment in rectal cancer with a two-protein immunohistochemical score derived from stromal gene-profiling. *Ann Oncol* **28**, 2160-2168, doi:10.1093/annonc/mdx293 (2017).
